# Isolation and Identification of Emerging Equine Encephalosis Virus Serotype 6 in Israel, 2023

**DOI:** 10.3390/pathogens15060571

**Published:** 2026-05-27

**Authors:** Natalia Golender, Zvia Mildenberg, Michel Bellaiche, Bernd Hoffmann

**Affiliations:** 1 Kimron Veterinary Institute, Bet Dagan 5025001, Israel; zviami@moag.gov.il (Z.M.); michelbe@moag.gov.il (M.B.); 2 Institute of Diagnostic Virology, Friedrich-Loeffler-Institut, 17493 Greifswald-Insel Riems, Germany; bernd.hoffmann@fli.de

**Keywords:** *Reoviridae*, *Orbivirus*, equine encephalosis virus, horse, donkey, descriptive epidemiology, sequencing, phylogeny

## Abstract

Equine encephalosis (EE) is an arthropod-borne viral disease resembling a mild form of African horse sickness disease, which affects all species of equids. Previously, only EEV serotype 4 (EEV-4) caused sporadic outbreaks of the disease in Israel. In the summer of 2023, EE was clinically diagnosed in horses and donkeys in Israel. For laboratory confirmation, tissue cultures were inoculated with whole-blood samples obtained from sick animals. Five EEV isolates were successfully recovered in tissue and confirmed by conventional RT-PCR and Sanger sequencing of several viral genes. BLAST analysis of genome segment 2 revealed that the isolates belonged to serotype 6. Full-genome sequencing of one representative strain and subsequent phylogenetic analysis demonstrated a close relationship to South African and Indian EEV strains, suggesting possible epidemiological links that warrant further investigation. This observation can indirectly point to a broader geographic circulation of EEV than was previously thought.

## 1. Introduction

Equine encephalosis (EE) is a noncontagious viral disease of equids caused by equine encephalosis virus (EEV). EEV (species *Orbivirus betaequi*) belongs to the genus *Orbivirus* in the family *Sedoreoviridae*, similarly to African horse sickness virus (AHSV), bluetongue virus (BTV), and epizootic hemorrhagic disease virus (EHDV) [1,2]. Like other orbiviruses, the EEV genome contains 10 linear double-stranded (ds) RNA segments (Seg-1 to Seg-10), which collectively encode seven structural proteins (VP1 to VP7) and five non-structural proteins (NS1 to NS5) [3,4,5]. EEV is classified into multiple serotypes based on antigenic and genetic variation in the viral outer capsid protein (VP2). Historically, seven EEV serotypes were identified in Southern Africa based on serological analyses [6].

EE can result in a wide variety of clinical signs in horses and is associated with changes in hematological variables that are similar to the milder forms of African horse sickness (AHS), and the two diseases can be easily confused [1,7]. Pyrexia, tachycardia, and tachypnoea are the most common clinical signs associated with EE. Hematological evaluation appears valuable in EE cases, with leukopenia, lymphopenia, and thrombocytopenia commonly observed [1]. EE is generally subclinical in donkeys and zebras, although occasional clinical manifestations have also been reported [1].

EEV is transmitted by *Culicoides* midges [8,9,10]. There is evidence of EE circulation in South Africa, East and West Africa, the Middle East, and India [11,12,13,14].

The first detection of EEV (serotype 4) in Israel was recorded in 2008–2009 and was mostly characterized by fever. However, symptoms such as depression, anorexia, edema, muscle pain, stiffness, generalized weakness, cough, and in some cases, nasal discharge and conjunctivitis were also observed in affected horses [15,16,17]. The disease spread throughout the country, and approximately 80% of the horse population was affected. The disease affected all breeds, ages, and sexes. Ninety percent of horses recovered from the disease without additional complications, and no deaths were reported [17].

In the summer–autumn period of 2023, EE was again clinically diagnosed in Israeli horses and donkeys. The aim of this study was to isolate, identify, and genetically characterize the EEV strain associated with the 2023 outbreak in Israel.

## 2. Materials and Methods

### 2.1. Field Samples

Whole blood samples from symptomatic horses and donkeys were collected with EDTA anticoagulant (ethylenediaminetetraacetic acid) and were submitted for laboratory diagnostics to the Virology Department of the Kimron Veterinary Institute (KVI), Israel.

### 2.2. Virus Isolation (VI)

Red blood cells, together with the buffy coat, were washed three times with PBS (infectious material, IM). IM was disrupted with sterile double-distilled water at a ratio of 1:10. Two types of tissue culture cells were used for virus isolation (VI): Vero (African green monkey kidney epithelial cells; source—ATCC) and BHK-BSR cells (baby hamster kidney cells, clone BSR), kindly provided by Prof. Eran Bachrach, Tel Aviv University, Israel.

One-day-old monolayers of these tissue cultures were incubated with IM for two hours in a cell incubator at 37 °C and then washed twice with PBS. Thereafter, maintenance medium, consisting of Dulbecco’s Modified Eagle’s Medium (DMEM, BIOWEST, Beit Haemek, Israel) supplemented with 2% FBS (fetal bovine serum, Biowest, Nuaillé, France), 1% tryptose phosphate broth (Sigma-Aldrich, St. Louis, MO, USA), and 1% penicillin–streptomycin (10,000 U/mL, BIOWEST, Beit Haemek, Israel), was added.

Infected tissue cultures were incubated in a cell incubator at 37 °C and observed daily for cytopathic effect (CPE) for 4–7 days, depending on cell condition. The supernatant was collected when 80–90% CPE was observed, followed by examination for EEV using conventional RT-PCR.

### 2.3. Nucleic Acid Extraction and RT-PCR Assays

Ribonucleic acid (RNA) was extracted from tissue culture supernatants and field samples using the MagMAX™ CORE Nucleic Acid Purification Kit (Thermo Fisher Scientific, Austin, TX, USA) or the IndiMag Pathogen Kit (Indical Bioscience, Leipzig, Germany), following the manufacturers’ instructions. Initial EEV RNA detection was performed using the OneStep RT-PCR Kit (Qiagen, Hilden, Germany), based on segments (Seg) 7 and 10. Primers used for the reactions are presented in Table 1. For EEV serotyping, pairs of primers were developed for all seven serotypes (only primers specific for EEV-6 are presented in Table 1).

After confirmation of EEV by sequencing, all collected and submitted samples were tested by real time RT-qPCR using the system, published by Rathogwa [18], Table 1, while the AgPath-ID™ One-Step RT-PCR Kit was used for this purpose (Life Technologies, Austin, TX, USA). Cut-off value for all assays was a cycle threshold (Ct) of 40.

### 2.4. Whole Genome Sequencing (WGS) and Phylogenetic Analyses

Due to identical partial sequences of Seg-2, -7, and 10, one strain was chosen for WGS. High-throughput sequencing (HTS) was carried out using the sequence-independent single-primer amplification (SISPA) approach [19], following the procedure described by Ries et al. [20] for double-stranded cDNA preparation. The resulting cDNA was submitted to Eurofins Genomics (Ebersberg, Germany) for genome sequencing on the Illumina platform. The resulting nucleotide (nt) sequences were assembled and aligned pairwise using Geneious Prime (version 2025.1, Biomatters, Auckland, New Zealand).

Alignment of nt sequences was done with BioEdit (https://bioedit.software.informer.com/7.2/, accessed on 12 February 2020). Phylogenetic trees were constructed using MEGA X software [21]. For all phylogenetic analyses, the maximum likelihood (ML) method and the Tamura–Nei model were applied.

## 3. Results

### 3.1. Investigation of Field Samples

A total of twenty-four horse and three donkey whole blood samples were received and tested during October 2023–May 2024. Twenty-two field horse and two donkey samples were positive in real-time RT-qPCR. Data on tested animals, geographic location, date of sampling, and clinical signs are shown in Table 2.

### 3.2. Clinical and Epidemiological Follow-Up

**Figure 1:**
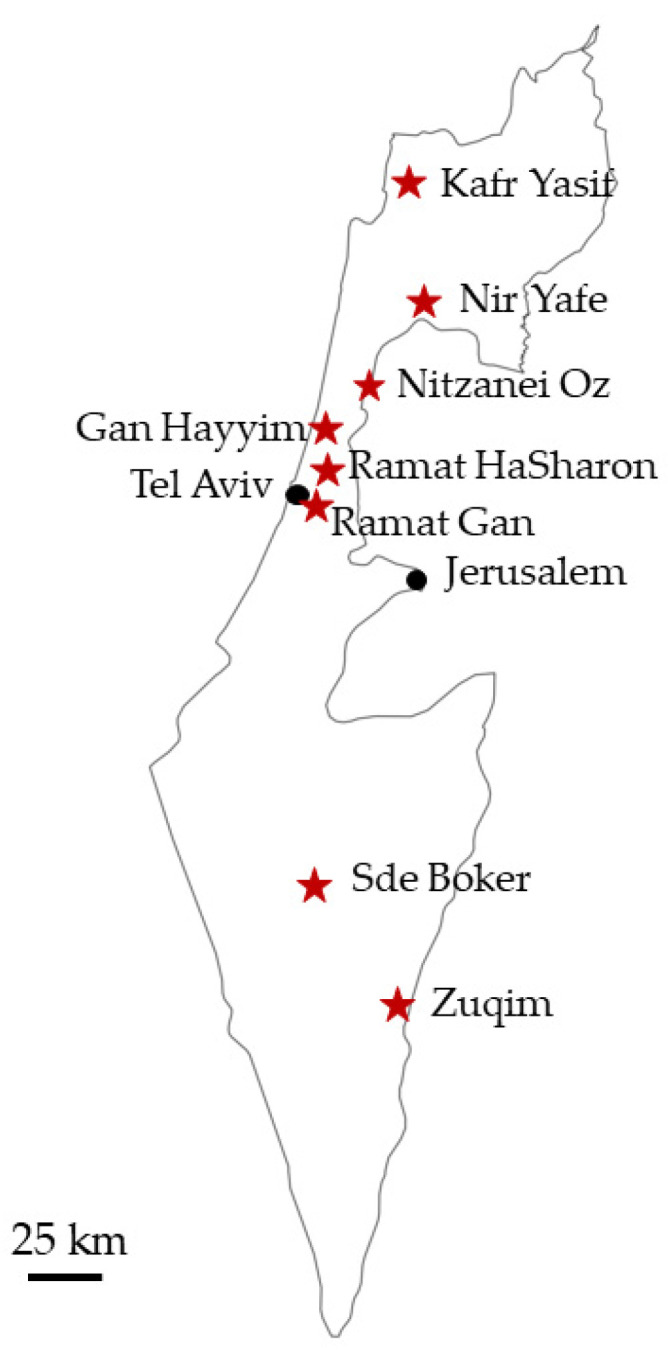
Red stars—geographic locations where EEV was confirmed in symptomatic horses.

First reports of febrile disease in horses were received at the beginning of September 2023. Tested field samples collected in October–November 2023 revealed the spread of EEV-6 in most of the territory of Israel (Figure 1 and Table 2). The percentage of clinically affected animals in farms where EEV-6 was confirmed was 53.33–100%. The geographic location of farms where samples from symptomatic horses were collected, and EEV consequently was confirmed, is shown in Figure 1.

### 3.3. Virus Isolation (VI)

Ten randomly chosen EDTA blood samples of potentially affected horses were used for infection of tissue cultures (Table 2). Five strains were isolated in both Vero and BSR cells at first passage. Cytopathic effect (CPE) was observed from day 3 after infection. An additional passage was performed for all used samples, where CPE was observed at passage 1 (for CPE confirmation), and without CPE. No additional strains were isolated at passage 2.

EEV was successfully isolated from some of the samples with RT-qPCR Ct values up to 30, showing clear results in the VP7 conventional PCR assay (sample 12). However, some samples with Ct values lower than 30 showed negative VI results and equivocal results in VP7 conventional PCR, which may indicate the inactivation of viral particles and/or degradation of the virus and its RNA (Table 2).

### 3.4. EEV Detection by RT-PCRs

To confirm the clinical diagnosis, all original blood samples, collected at October 23, 2023, were tested for a fragment of VP7 in conventional RT-PCR, while 10 of them were estimated as positive, and sent for Sanger sequencing (Hy Laboratories Ltd., Rehovot, Israel). Additionally, five PCR fragments of Seg-10 were amplified from viruses successfully propagated in cell cultures and also subjected to Sanger sequencing. Sequenced fragments confirmed a relationship to EEV and had up to 100% identity with each other by segments 7 and 10 (Table 2). All five virus isolates (strains) were tested using Seg-2 serotype-specific PCR systems for each serotype (serotypes 1–7), and all five virus isolates were positive in the EEV-6-specific system. Subsequently, two of the Seg-2 PCR fragments were also sequenced. BLASTn analysis of both partial Seg-2 sequences revealed a 95.81% match with the South African EEV-6 isolate Potchefstroom from 1991.

### 3.5. Whole Genome Sequencing and BLAST Analyses

Coding regions of all ten segments were assembled and submitted to the GenBank, NCBI website (accession numbers (acc. num.) PX980498-PX980507).

Results of BLASTn analyses are shown in Table 3. Only the closest EEV strain is shown in Table 3 for every genome segment, except Seg-9, due to identical results of nt identity of three different EEV strains with Israeli EEV-6 by BLASTn analyses.

Results of BLASTp analyses is shown in Table 3. Only the closest EEV strain is shown in Table 4 for every genome segment, except Seg-8 and -9, due to identical results of the two and three different EEV strains, respectively, with Israeli EEV-6 by BLASTp analyses.

Analyzing the presented data, Israeli EEV-6 had the closest nt identity with different South African EEV strains by seven in ten genome segments, belonging to different serotypes. The rest of the genes had the closest identity with the Indian EEV-1 strain 88403, isolated in 2008. Considering amino acid (aa) identity, eight or nine genome segments have the closest identity with different South African EEV strains, with seven of the ten genome segments belonging to different serotypes (Seg-8 of Israeli EEV-6 had the same identity with two different South African and Indian EEV-1 strain 88403). The remaining one or two genome segments had the closest identity with the Indian EEV-1 strain 88403.

### 3.6. Phylogenetic Analyses

#### 3.6.1. Analysis of Serotype-Defining Outer Protein-Coding Genes (Seg-2 and Seg-6)

**Figure 2:**
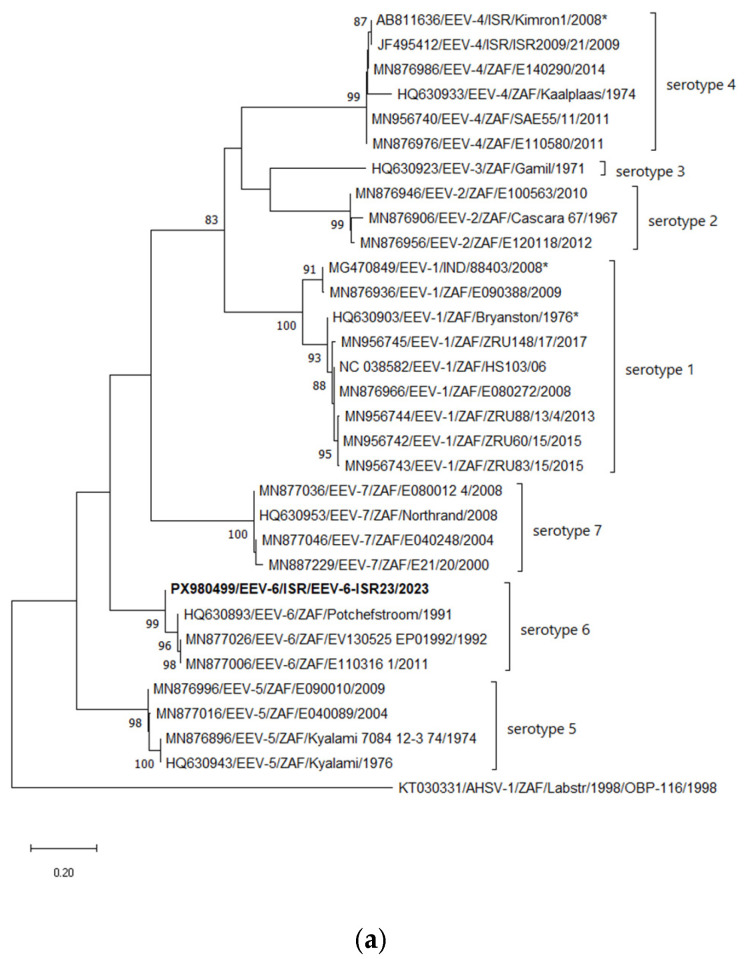

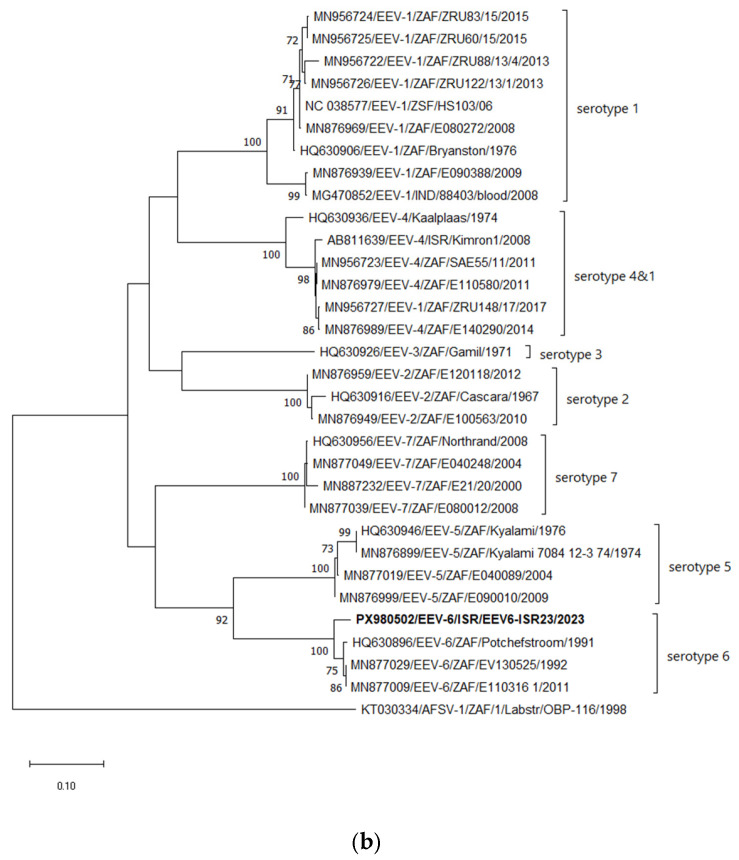
Phylogenetic tree of segment 2 of Israeli EEV-6 isolated in 2023. (**a**) Phylogenetic tree of segment 2 and global EEV strains. (**b**) Phylogenetic tree of segment 6 and global EEV strains. The Israeli EEV-6 strain is shown in bold. The phylogeny was inferred using the Maximum Likelihood method and the Tamura–Nei model method. The percentage of replicate trees in which the associated taxa clustered together in the bootstrap test (1000 replicates) is shown next to the branches. Only bootstrap values above 70% are displayed. Reference strains included all available strains from GenBank, excluding duplicate sequences sharing nearly 100% nt identity. Viruses were identified by accession number/serotype/location/isolate/year. African horse sickness serotype 1 strain OBP-116 was used as an outgroup for both phylogenetic trees; *—corrected serotypes for several EEV strains.

According to the phylogenetic analysis of Seg-2, similarly to the BLASTn analyses, the Israeli strain EEV-6-ISR23 clusters with all South African EEV-6 strains, while the Potchefstroom strain isolated in 1991 had the closest relationship (Figure 2a). Considering Seg-6, coding for the VP5 protein, Israeli strain EEV-6-ISR23 clusters with all South African EEV-6 strains, forming a monophyletic branch (Figure 2b). Phylogenetic incongruences between segments may suggest possible reassortment events among EEV strains, although further analyses are required to confirm this hypothesis. (Figure 2b).

#### 3.6.2. Phylogenetic Analyses of Internal Genes (Seg-1, -3, -4, -5, -7, -8, -9, and -10)

According to phylogenetic analysis of Seg-1, the Israeli EEV-6 strain EEV6-ISR23 forms a cluster with several South African strains of types EEV-1, -5 and -7 that were isolated between 2004 and 2013 (Figure S1a). Interestingly, the phylogenetic trees of Seg-3 showed the formation of a monophyletic clade by the Israeli EEV-6 strain, which likely shares a common ancestor with several South African EEV strains isolated between 1992 and 2017. This is the only gene for which no relationship with the Indian EEV-1 strain 88403, isolated in 2008, was detected (Figure S1b). Phylogenetic tree of Seg-4, -5, -7, -8, -10 revealed close identity and clustering with both South African and Indian EEV strains, isolated between 2008 and 2017 (Figure S1c–f,h). Considering Seg-9, Israeli EEV-6 strain EEV6-ISR23 forms a monophyletic branch, having a less significant relationship both with South African and Indian strains, isolated between 1992 and 2017 (Figure S1g).

## 4. Discussion

Over the past two decades, the Israeli livestock population has been affected by arboviruses that had not previously been detected in this region [22]. Moreover, the appearance of new pathogenic arboviruses in the region has also been observed in some other countries of the Mediterranean region and even in Eastern European countries, similarly to Israel. Thus, possible common ancestors have been suggested for EHDV-6, BTV-3, -4, -8, -12 and, lastly, BTV-5 [23,24,25,26,27,28], which were identified in North Africa and in many European countries.

Notably, the appearance of many different serotypes and strains of BTV has been reported previously in Israel and, more recently, in some European countries [24,26,27,28]. Genetic analysis of BTV serotypes commonly found in Israel and Europe revealed that the outbreaks in these countries were caused by different strains. It can therefore be concluded that, whilst there was no direct transmission of the viruses between the regions, they do share a common ancestry [24].

Most of these recently identified Israeli strains have a segmented genome. Shuni virus (SHUV) [29], numerous BTV serotypes (-1, -3, -4, -5, -6, -8, -9 and -11) [24], as well as EHDV-1 and -6 [23], showed a clear African origin and were strongly reassorted with local Israeli strains, suggesting their concurrent circulation. In contrast to BTV, EHDV and SHUV, no relationship was revealed between previously identified Israeli EEV-4 from 2008 to 2009 and the recently identified EEV-6, probably indicating the absence of co-circulation of these viruses in the region.

In contrast to orbiviruses of ruminants, which are widely distributed in many parts of the world [30], orbiviruses of equids have been reported mostly on the African continent. Only a few outbreaks of AHS have been registered outside Africa, mainly in regions adjacent to the African continent [31]. Considering EEV, outside Africa, it was detected in 2008 in India and twice in Israel (in 2008–2009 and 2023) [13,15].

Based on newly published EEV sequences from recent years, inconsistencies have been noted in three EEV strains. The phylogenetic analyses presented here justify a revised serotyping of these strains. Two strains previously classified as EEV-4 (Indian strain 88403, acc. num. MG480849, and South African strain Bryanston, acc. num. HQ630903) should be reassigned to EEV-1, while the Israeli EEV-3 strain (acc. num. AB811636) should be reassigned to EEV-4.

According to BLAST and phylogenetic analyses, both the Israeli EEV6-ISR23 and the Indian EEV-1 strain 88403, isolated in 2008, likely originate from the same group of African EEV ancestor strains, which belong to various EEV serotypes and mostly date from 2004 to 2017 (including the most recently published South African strains). This observation may also indirectly suggest a wider distribution of EEV, which requires broader investigations and filling the gaps in knowledge about the actual EEV distribution.

According to our analysis of all internal genes of EEV, several distinct groups of EEV exist that are not related to the Indian and Israeli strains mentioned above. Notably, there is no relationship, based on internal genes, between the recent Israeli EEV-6 and the Indian EEV-1 strain from 2008 and the previously identified Israeli EEV-4 from 2008. Based on these analyses, we can presume that there were at least two independent introductions of EEV into Israel and that there was probably no circulation of the previously identified Israeli EEV-4 in the region.

At present, assessment of a more accurate EE distribution and the origin of recently identified EEV strains is not feasible due to scarce EEV identification in the affected areas (mainly the African continent) and limited publicly available data.

According to the publication by Venter et al. [9], EEV-1 (Bryanston strain) successfully infects *C. imicola*, suggesting that this species may be able to transmit the virus to susceptible hosts, while no virus replication was demonstrated in 102 *C. nivosus* specimens. It was suggested that not all *Culicoides* species are equally susceptible to Bryanston virus infection [9].

Notably, according to a recent review article [32], *C. imicola* has been recorded in 50 countries, including the entire African continent, Madagascar, some regions of Asia and Southern Europe, where transmission of the virus by this vector is potentially possible. Regarding Israel, sixty *Culicoides* species have been recorded, while *C. imicola* is considered the dominant species [33]. However, no investigations on EEV detection in *Culicoides* spp. or vector competence studies on *Culicoides* species abundant in Israel have been performed, which makes the estimation of their role in EEV transmission in the region difficult.

Lastly, the incidence of new arboviruses pathogenic to farm animals is higher than ever before, a trend that may be linked to climate change and the spread of vectors or infected animals into new areas [34]. The identification of a new EEV-6 strain in Israel in 2023 can be interpreted as a sign of an increased risk of new arboviruses emerging in equines in European countries as well, analogous to the situation with arboviruses in ruminants.

## 5. Conclusions

The Israeli EEV-6 strain EEV6-ISR23, isolated in 2023, and the Indian EEV-1 strain 88403 probably originated from the same group of African EEV strains.

No close phylogenetic relationship was identified between the previously reported Israeli EEV-4 and the current EEV-6 strain, suggesting that these viruses may represent separate introduction events into Israel.

## Figures and Tables

**Table 1:** List of probes/primers used for identification and sequencing of equine encephalosis virus.

Segment/Serotype	Name	Sequence of Oligo 5′–3′	Length of Product	Source
10/all	EEV-NS3-1F	GTT AAG TTT CTG CGC CAT GT	760	[16]
	EEV-NS3-741R	GTA ACA CGT TTC CGC CAC G		[16]
7/all	EEV-VP7-28F	GATAGCGGCTAGAGCCCTTTC	343	[18]
	EEV-VP7-350R	CTG CGT CTC TCC TGT CAC CCT		this study
2/6	EEV6-VP2-1F	GTT TAA TTG AGC GTG GAG ATG G	596	this study
	EEV6-VP2-575R	CGA TGC TTT GAT TCA CGC ACT A		this study
7/all	EEV-VP7-28F	GATAGCGGCTAGAGCCCTTTC	78	[18]
	EEV-VP7-85-106R	AACTTGAGGAGCCATRGTAGCT		[18]
	EEV-54-PROBE	FAM-TAAGAGCATGTGTTACTGC-MGB		[18]

**Table 2:** Information on symptomatic animals, geographic location of affected farms, and laboratory tests.

Animal No.	Sampling Date	Place	Species	Sex	Age Category	Clinical Signs	qRT-PCR (Ct Value)	Sanger Seq	EEV-6 RT-PCR	VI
1	23 October 2023	Nitzanei Oz	horse	no data	adult	fever, inappetence, colic, limb edema	25.72	VP7, NS3-pos	n.p.	pos
2	23 October 2023	Nitzanei Oz	horse	no data	adult	fever, inappetence, colic, limb edema	30.62	VP7-equivocal	n.p.	n.p.
3	23 October 2023	Nitzanei Oz	horse	no data	adult	fever, inappetence, colic, limb edema, weakness	33.85	VP7-equivocal	n.p.	n.p.
4	23 October 2023	Nitzanei Oz	horse	no data	adult	fever, inappetence, colic, limb edema	25.44	VP7, NS3-pos	n.p.	pos
5	23 October 2023	Nitzanei Oz	horse	no data	adult	fever, inappetence, colic, limb edema, weakness	37.81	VP7-equivocal	n.p.	n.p.
6	23 October 2023	Nitzanei Oz	horse	no data	adult	fever, inappetence, colic, limb edema, weakness	31.86	VP7-equivocal	n.p.	n.p.
7	23 October 2023	Nitzanei Oz	horse	no data	adult	fever, inappetence, colic, limb edema, weakness	22.75	VP7-pos	n.p.	neg
8	23 October 2023	Gan Hayyim	horse	no data	adult	fever, inappetence, weakness and fatigue	28.14	VP7-equivocal	n.p.	n.p.
9	23 October 2023	Gan Hayyim	horse	no data	adult	fever, inappetence, weakness and fatigue	neg	VP7-equivocal	n.p.	n.p.
10	23 October 2023	Gan Hayyim	horse	no data	adult	fever, inappetence, weakness and fatigue	31	VP7-pos	n.p.	neg
11	23 October 2023	Gan Hayyim	horse	no data	adult	fever, inappetence, weakness and fatigue	24	VP7-pos	n.p.	neg
12	23 October 2023	Sde Boker	horse	no data	adult	a high fever, inappetence	30.04	VP7, NS3-pos	n.p.	pos *
13	23 October 2023	Sde Boker	horse	no data	adult	a high fever, inappetence	31.79	VP7 pos	n.p.	neg
14	23 October 2023	Nir Yafe	horse	no data	adult	a high fever, inappetence	27.18	VP7, NS3-pos	n.p.	pos
15	23 October 2023	Nir Yafe	horse	no data	adult	fever, inappetence, weakness	23.15	VP7 pos	n.p.	neg
16	23 October 2023	Ramat Hasharon	horse	no data	adult	fever, inappetence, colic, limb edema, weakness	28.13	VP7-equivocal	n.p.	n.p.
17	23 October 2023	Ramat Hasharon	horse	no data	adult	fever, inappetence, colic, limb edema, weakness	28.31	VP7-equivocal	n.p.	n.p.
18	23 October 2023	Ramat Hasharon	horse	no data	adult	fever, inappetence, colic, limb edema, weakness	25.53	VP7 pos	n.p.	EEV pos
19	23 October 2023	Ramat Hasharon	horse	no data	adult	fever, inappetence, colic, limb edema, weakness	31.76	VP7-equivocal	n.p.	n.p.
20	23 October 2023	Zuqim	horse	no data	adult	a high fever, inappetence	31.94	VP7-equivocal	n.p.	n.p.
21	23 October 2023	Zuqim	horse	no data	adult	a high fever, inappetence	33.36	VP7-equivocal	n.p.	n.p.
22	21 November 2023	no data	horse	male	adult	fever, diarrhea	27.38	n.p.	pos	n.p.
23	21 November 2023	Kafr Yasif	horse	female	adult	fever, diarrhea	28.09	n.p.	pos	n.p.
24	26 November 2023	Kafr Yasif	donkey	male	adult	no data	25.45	n.p.	pos	n.p.
25	26 November 2023	Kafr Yasif	donkey	female	adult	fever, diarrhea	27.11	n.p.	pos	n.p.
26	10 April 2024	no data	horse	no data	adult	no data	neg	n.p.	n.p.	n.p.
27	1 May 2024	no data	donkey	no data	adult	no data	neg	n.p.	n.p.	n.p.

* WGS of the virus isolate was performed; n.p.—not performed; pos—positive; neg—negative; a high fever—body temperature ≥ 40 °C.

**Table 3:** BLASTn analysis of Israeli equine encephalosis virus strain EEV-6-ISR23 compared with global EEV strains.

Segment	Identity (%)	Accession Number/Serotype/Strain/Year	Country of Isolation
1	98.25	MN877015/EEV-5/Midrand-E040089/2004	South Africa
2	95.04	HQ630893/EEV-6/Potchefstroom/1991	South Africa
3	98.47	MN877047/EEV-7/Steynsburg-E040248/2004	South Africa
4	97.7	MG470851/EEV-1/88403/2008	India
5	98.84	MG470865/EEV-1/88403/2008	India
6	95.27	MN877029/EEV-6/E130525_EP01992/2013	South Africa
7	98.53	MG470864/EEV-1/88403/2008	India
8	98.88	MN876943/EEV-1/Ingogo-E090388/2009	South Africa
9	97.25	MN876992/EEV-4/Rustenburg-E140290/2014	South Africa
9	97.25	MN877010/EEV-6/E110316_1/2011	South Africa
9	97.25	HQ630957 /EEV-7/Northrand/2008	South Africa
10	95.96	HQ630891/EEV-6/Potchefstroom/1991	South Africa

**Table 4:** BLASTp analysis of Israeli equine encephalosis virus strain EEV-6-ISR23 compared with global EEV strains.

Segment/ Protein	Identity (%)	Accession Number/Serotype/Strain/Year	Country of Isolation
1/VP1	99.46	MN877015/EEV-5/Midrand-E040089/2004	South Africa
2/VP2	96.44	HQ630893/EEV-6/Potchefstroom/1991	South Africa
3/VP3	99.78	HQ630914/EEV-1/Cascara/1967	South Africa
4/VP4	98.45	MN876998/EEV-5/E090010/2009	South Africa
5/NS1	99.45	MG470855/EEV-1/88403/2008	India
5/NS1	99.45	MN876982/EEV-4/Norvalspont-E110580/2011	South Africa
6/VP5	99.03	HQ630896/EEV-6/Potchefstroom/1991	South Africa
7/VP7	99.71	HQ630908/EEV-1/Bryanston/1976	South Africa
8/NS2	99.45	MG470856/EEV-1/88403/2008	India
8/NS2	99.45	NC_038583/EEV-1/HS103/06	South Africa
8/NS2	99.45	MN876983/EEV-4/Norvalspont-E110580/2011	South Africa
9/VP6	96.19	MN876990/EEV-4/Rustenburg-E140290/2014	South Africa
9/VP6	96.19	MN956710/EEV-1/ZRU148/17/2017	South Africa
9/VP6	96.19	MN877010/EEV-6/Everton-E110316_1/2011	South Africa
10/NS3	98.75	AY115873/EEV-6/E92/2000	South Africa

## Data Availability

The original contributions presented in this study are included in the article/Supplementary Materials. Further inquiries can be directed to the corresponding author.

## Supplementary Materials

The following supporting information can be downloaded at: https://www.mdpi.com/article/10.3390/pathogens15060571/s1, Figure S1: Phylogenetic trees of Israeli EEV-6 of internal genes and global strains.
