# Supplementary material for: Isolation and Identification of Emerging Equine Encephalosis Virus Serotype 6 in Israel, 2023
Source: Pathogens. 2026 May 27;15(6):571. doi: 10.3390/pathogens15060571 (PMC13304881; doi:10.3390/pathogens15060571)
Supplement: Supplementary file 1 [file pathogens-15-00571-s001.zip › pathogens-4324182-supplementary.pdf]

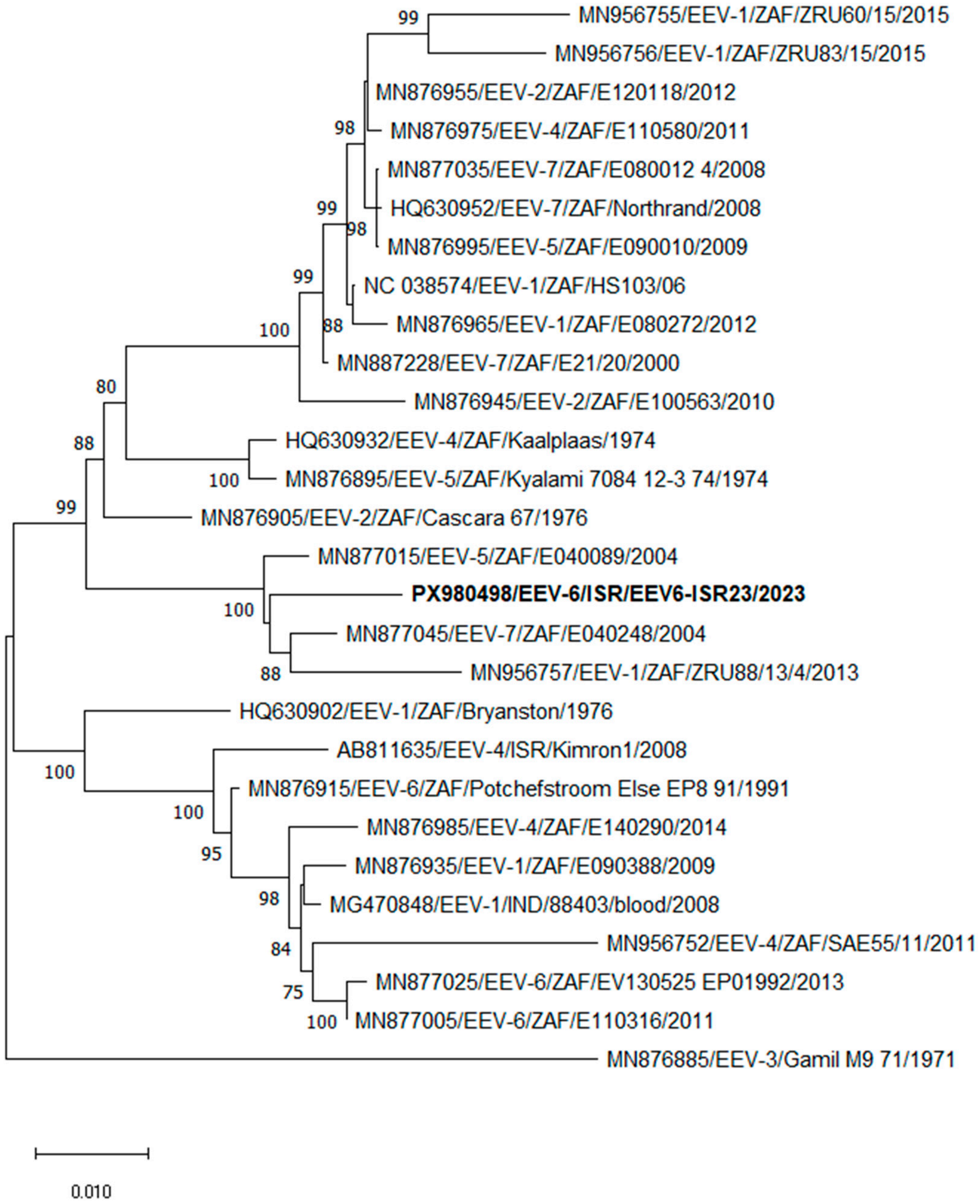

MN956755/EEV-1/ZAF/ZRU60/15/2015
MN956756/EEV-1/ZAF/ZRU83/15/2015
MN876955/EEV-2/ZAF/E120118/2012
MN876975/EEV-4/ZAF/E110580/2011
MN877035/EEV-7/ZAF/E080012 4/2008
HQ630952/EEV-7/ZAF/Northrand/2008
MN876995/EEV-5/ZAF/E090010/2009
NC 038574/EEV-1/ZAF/HS103/06
MN876965/EEV-1/ZAF/E080272/2012
MN887228/EEV-7/ZAF/E21/20/2000
MN876945/EEV-2/ZAF/E100563/2010
HQ630932/EEV-4/ZAF/Kaalplaas/1974
MN876895/EEV-5/ZAF/Kyalami 7084 12-3 74/1974
MN876905/EEV-2/ZAF/Cascara 67/1976
MN877015/EEV-5/ZAF/E040089/2004
PX980498/EEV-6/ISR/EEV6-ISR23/2023
MN877045/EEV-7/ZAF/E040248/2004
MN956757/EEV-1/ZAF/ZRU88/13/4/2013
HQ630902/EEV-1/ZAF/Bryanston/1976
AB811635/EEV-4/ISR/Kimron1/2008
MN876915/EEV-6/ZAF/Potchefstroom Else EP8 91/1991
MN876985/EEV-4/ZAF/E140290/2014
MN876935/EEV-1/ZAF/E090388/2009
MG470848/EEV-1/IND/88403/blood/2008
MN956752/EEV-4/ZAF/SAE55/11/2011
MN877025/EEV-6/ZAF/EV130525 EP01992/2013
MN877005/EEV-6/ZAF/E110316/2011
MN876885/EEV-3/Gamil M9 71/1971
99
98
99
98
99
88
100
80
88
100
99
100
88
100
100
95
98
84
75
100
0.010

(a)

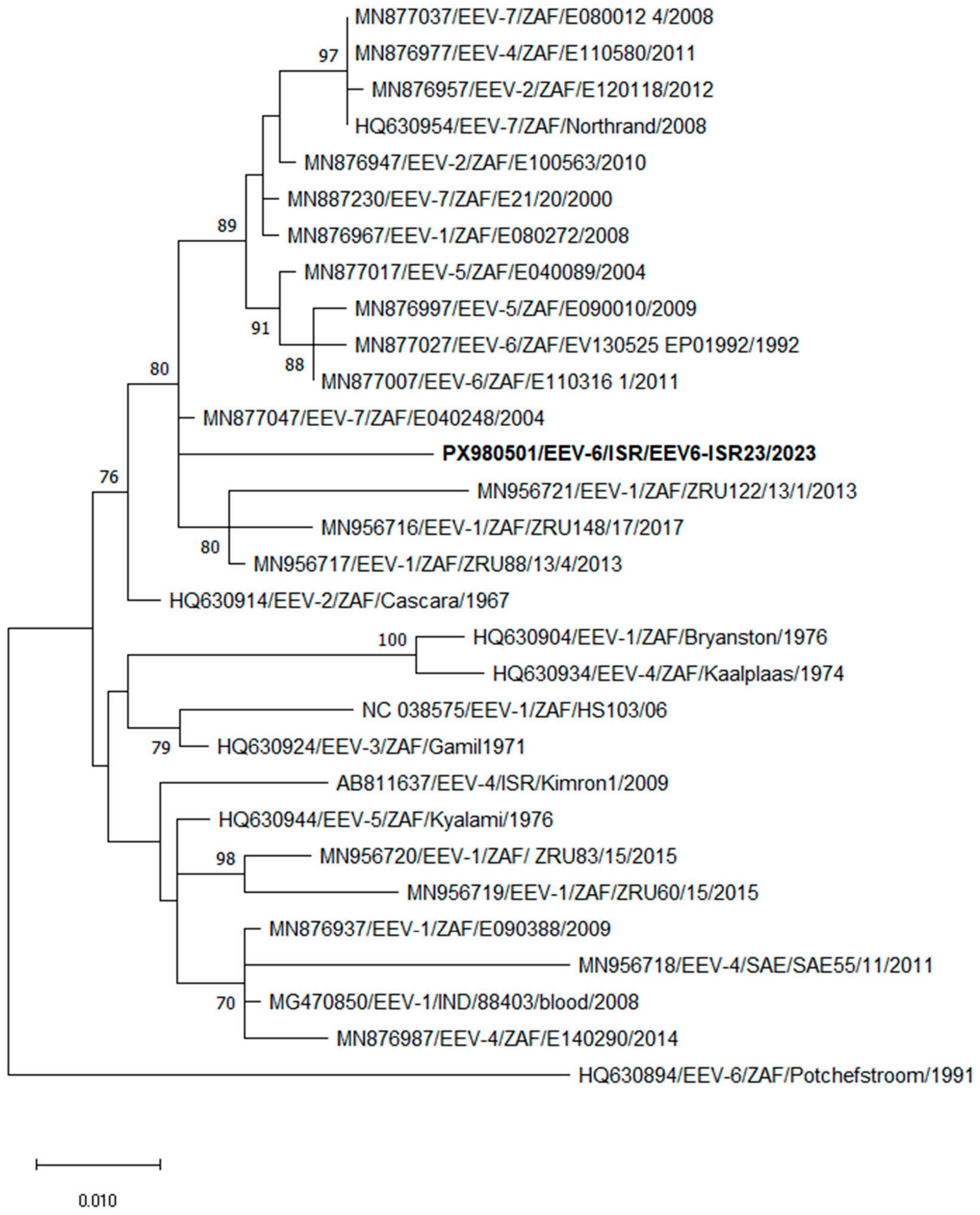
MN877037/EEV-7/ZAF/E080012 4/2008
97
MN876977/EEV-4/ZAF/E110580/2011
MN876957/EEV-2/ZAF/E120118/2012
HQ630954/EEV-7/ZAF/Northrand/2008
MN876947/EEV-2/ZAF/E100563/2010
MN887230/EEV-7/ZAF/E21/20/2000
89
MN876967/EEV-1/ZAF/E080272/2008
MN877017/EEV-5/ZAF/E040089/2004
MN876997/EEV-5/ZAF/E090010/2009
91
MN877027/EEV-6/ZAF/EV130525 EP01992/1992
88
MN877007/EEV-6/ZAF/E110316 1/2011
80
MN877047/EEV-7/ZAF/E040248/2004
PX980501/EEV-6/ISR/EEV6-ISR23/2023
76
MN956721/EEV-1/ZAF/ZRU122/13/1/2013
MN956716/EEV-1/ZAF/ZRU148/17/2017
80
MN956717/EEV-1/ZAF/ZRU88/13/4/2013
HQ630914/EEV-2/ZAF/Cascara/1967
100
HQ630904/EEV-1/ZAF/Bryanston/1976
HQ630934/EEV-4/ZAF/Kaalplaas/1974
NC 038575/EEV-1/ZAF/HS103/06
79
HQ630924/EEV-3/ZAF/Gamil1971
AB811637/EEV-4/ISR/Kimron1/2009
HQ630944/EEV-5/ZAF/Kyalami/1976
98
MN956720/EEV-1/ZAF/ ZRU83/15/2015
MN956719/EEV-1/ZAF/ZRU60/15/2015
MN876937/EEV-1/ZAF/E090388/2009
MN956718/EEV-4/SAE/SAE55/11/2011
70
MG470850/EEV-1/IND/88403/blood/2008
MN876987/EEV-4/ZAF/E140290/2014
HQ630894/EEV-6/ZAF/Potchefstroom/1991
0.010

(b)

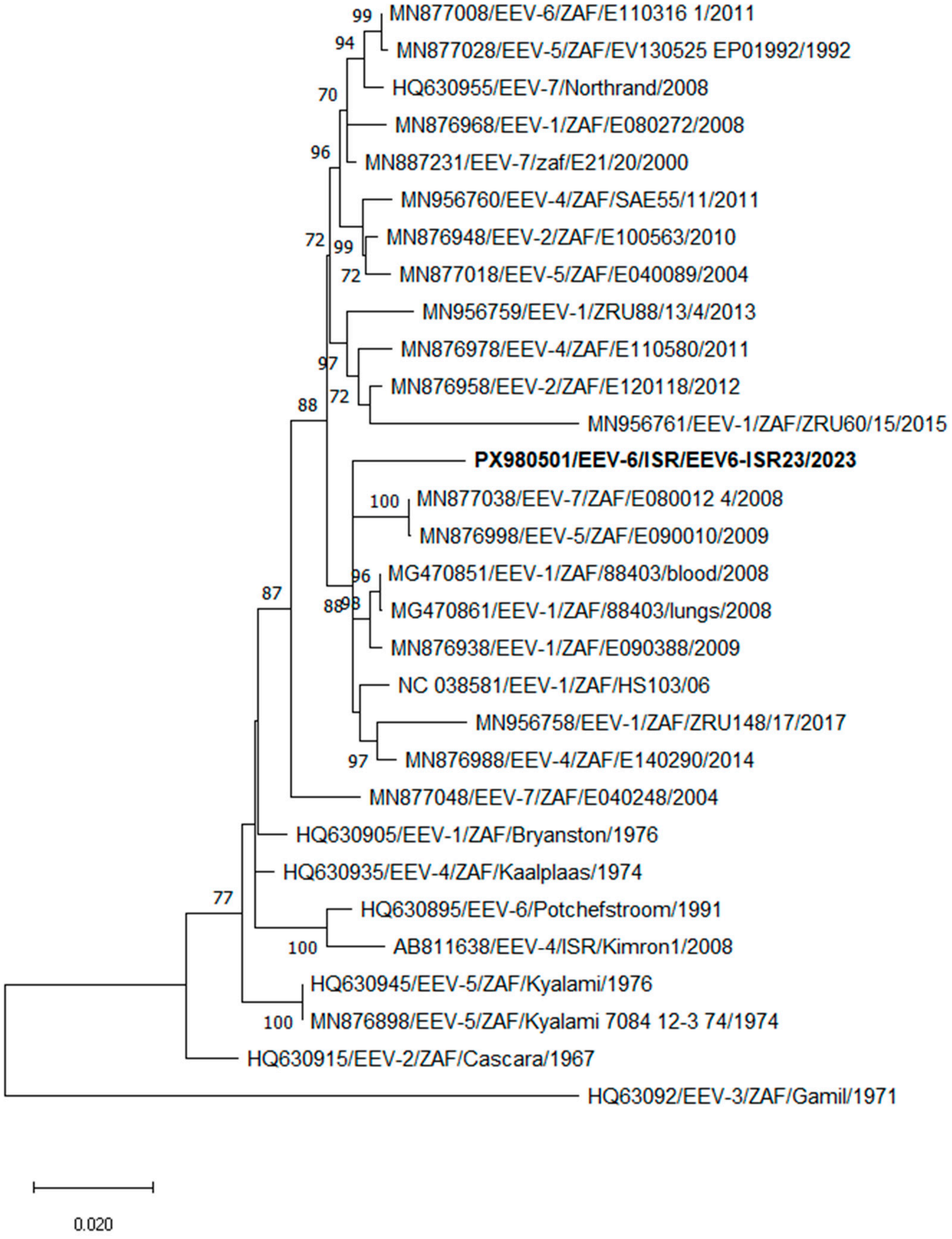
MN877008/EEV-6/ZAF/E110316 1/2011
MN877028/EEV-5/ZAF/EV130525 EP01992/1992
HQ630955/EEV-7/Northrand/2008
MN876968/EEV-1/ZAF/E080272/2008
MN887231/EEV-7/zaf/E21/20/2000
MN956760/EEV-4/ZAF/SAE55/11/2011
MN876948/EEV-2/ZAF/E100563/2010
MN877018/EEV-5/ZAF/E040089/2004
MN956759/EEV-1/ZRU88/13/4/2013
MN876978/EEV-4/ZAF/E110580/2011
MN876958/EEV-2/ZAF/E120118/2012
MN956761/EEV-1/ZAF/ZRU60/15/2015
PX980501/EEV-6/ISR/EEV6-ISR23/2023
MN877038/EEV-7/ZAF/E080012 4/2008
MN876998/EEV-5/ZAF/E090010/2009
MG470851/EEV-1/ZAF/88403/blood/2008
MG470861/EEV-1/ZAF/88403/lungs/2008
MN876938/EEV-1/ZAF/E090388/2009
NC 038581/EEV-1/ZAF/HS103/06
MN956758/EEV-1/ZAF/ZRU148/17/2017
MN876988/EEV-4/ZAF/E140290/2014
MN877048/EEV-7/ZAF/E040248/2004
HQ630905/EEV-1/ZAF/Bryanston/1976
HQ630935/EEV-4/ZAF/Kaalplaas/1974
HQ630895/EEV-6/Potchefstroom/1991
AB811638/EEV-4/ISR/Kimron1/2008
HQ630945/EEV-5/ZAF/Kyalami/1976
MN876898/EEV-5/ZAF/Kyalami 7084 12-3 74/1974
HQ630915/EEV-2/ZAF/Cascara/1967
HQ63092/EEV-3/ZAF/Gamil/1971
99
94
70
96
72
99
72
97
72
88
100
96
88
98
97
87
100
77
100
0.020

(c)

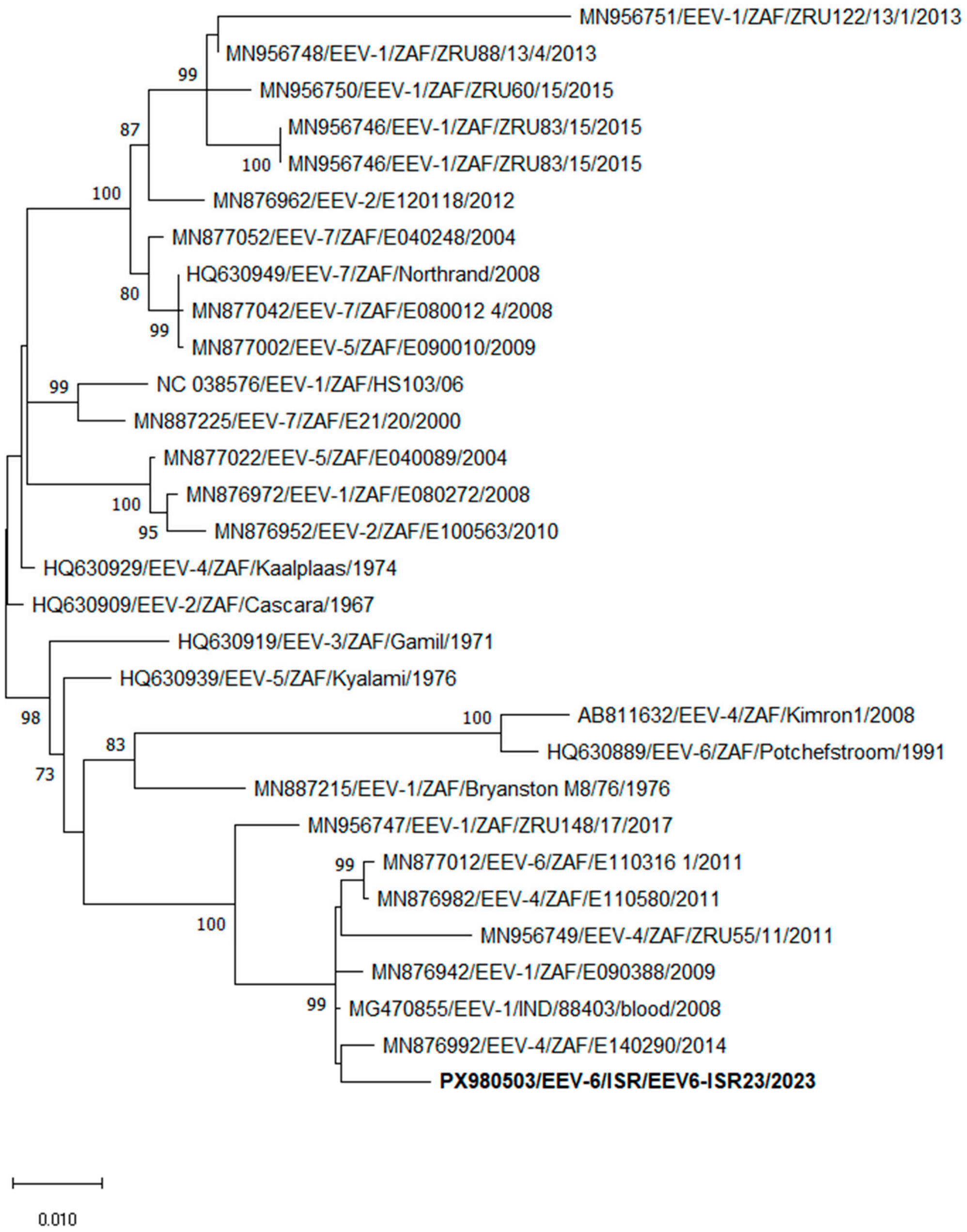
MN956751/EEV-1/ZAF/ZRU122/13/1/2013
MN956748/EEV-1/ZAF/ZRU88/13/4/2013
99
MN956750/EEV-1/ZAF/ZRU60/15/2015
87
MN956746/EEV-1/ZAF/ZRU83/15/2015
100
MN956746/EEV-1/ZAF/ZRU83/15/2015
100
MN876962/EEV-2/E120118/2012
MN877052/EEV-7/ZAF/E040248/2004
HQ630949/EEV-7/ZAF/Northrand/2008
80
MN877042/EEV-7/ZAF/E080012 4/2008
99
MN877002/EEV-5/ZAF/E090010/2009
99
NC 038576/EEV-1/ZAF/HS103/06
MN887225/EEV-7/ZAF/E21/20/2000
MN877022/EEV-5/ZAF/E040089/2004
MN876972/EEV-1/ZAF/E080272/2008
100
95
MN876952/EEV-2/ZAF/E100563/2010
HQ630929/EEV-4/ZAF/Kaalplaas/1974
HQ630909/EEV-2/ZAF/Cascara/1967
HQ630919/EEV-3/ZAF/Gamil/1971
HQ630939/EEV-5/ZAF/Kyalami/1976
100
AB811632/EEV-4/ZAF/Kimron1/2008
98
83
HQ630889/EEV-6/ZAF/Potchefstroom/1991
73
MN887215/EEV-1/ZAF/Bryanston M8/76/1976
MN956747/EEV-1/ZAF/ZRU148/17/2017
99
MN877012/EEV-6/ZAF/E110316 1/2011
MN876982/EEV-4/ZAF/E110580/2011
100
MN956749/EEV-4/ZAF/ZRU55/11/2011
MN876942/EEV-1/ZAF/E090388/2009
99
MG470855/EEV-1/IND/88403/blood/2008
MN876992/EEV-4/ZAF/E140290/2014
PX980503/EEV-6/ISR/EEV6-ISR23/2023
0.010

(d)

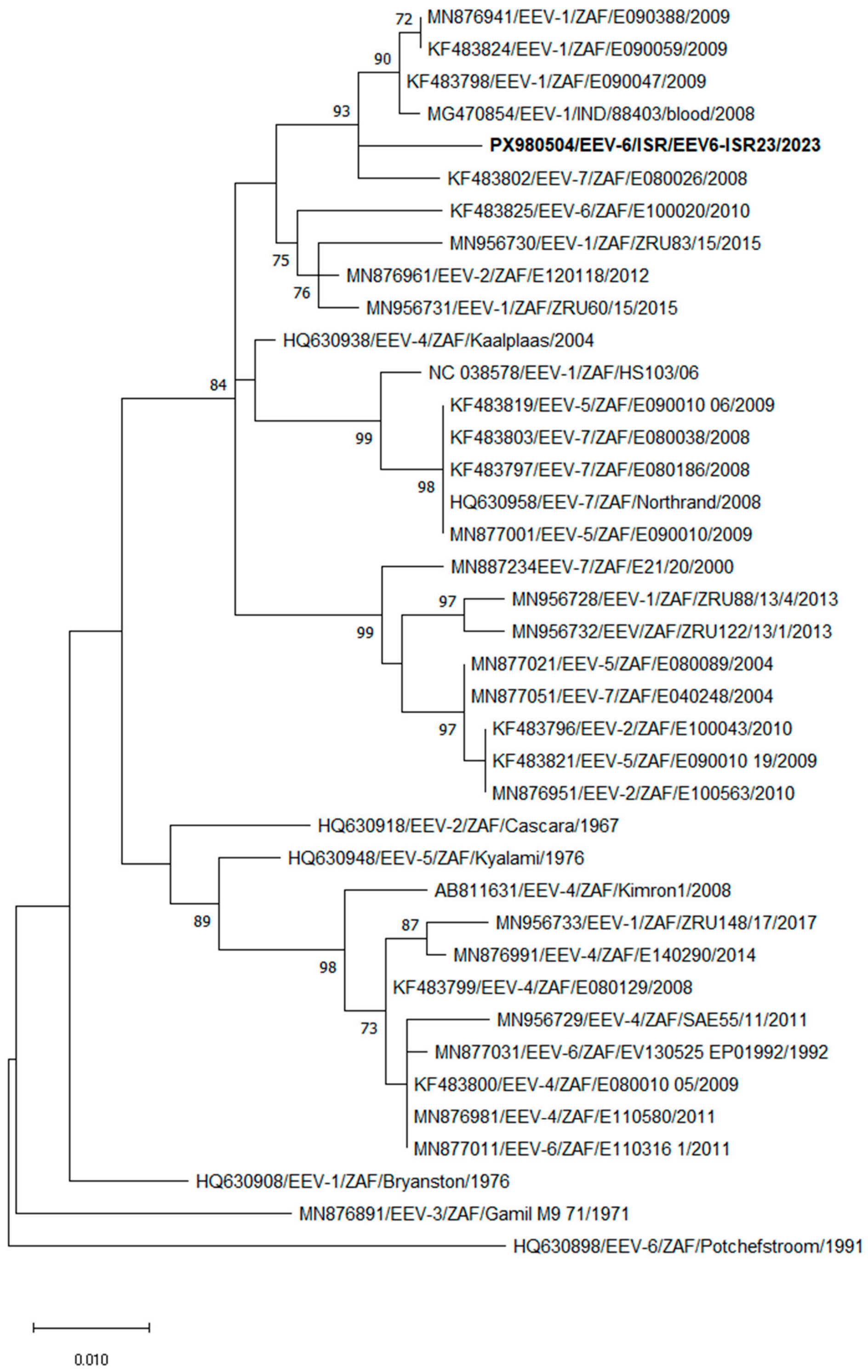
MN876941/EEV-1/ZAF/E090388/2009
KF483824/EEV-1/ZAF/E090059/2009
KF483798/EEV-1/ZAF/E090047/2009
MG470854/EEV-1/IND/88403/blood/2008
PX980504/EEV-6/ISR/EEV6-ISR23/2023
KF483802/EEV-7/ZAF/E080026/2008
KF483825/EEV-6/ZAF/E100020/2010
MN956730/EEV-1/ZAF/ZRU83/15/2015
MN876961/EEV-2/ZAF/E120118/2012
MN956731/EEV-1/ZAF/ZRU60/15/2015
HQ630938/EEV-4/ZAF/Kaalplaas/2004
NC 038578/EEV-1/ZAF/HS103/06
KF483819/EEV-5/ZAF/E090010 06/2009
KF483803/EEV-7/ZAF/E080038/2008
KF483797/EEV-7/ZAF/E080186/2008
HQ630958/EEV-7/ZAF/Northrand/2008
MN877001/EEV-5/ZAF/E090010/2009
MN887234EEV-7/ZAF/E21/20/2000
MN956728/EEV-1/ZAF/ZRU88/13/4/2013
MN956732/EEV/ZAF/ZRU122/13/1/2013
MN877021/EEV-5/ZAF/E080089/2004
MN877051/EEV-7/ZAF/E040248/2004
KF483796/EEV-2/ZAF/E100043/2010
KF483821/EEV-5/ZAF/E090010 19/2009
MN876951/EEV-2/ZAF/E100563/2010
HQ630918/EEV-2/ZAF/Cascara/1967
HQ630948/EEV-5/ZAF/Kyalami/1976
AB811631/EEV-4/ZAF/Kimron1/2008
MN956733/EEV-1/ZAF/ZRU148/17/2017
MN876991/EEV-4/ZAF/E140290/2014
KF483799/EEV-4/ZAF/E080129/2008
MN956729/EEV-4/ZAF/SAE55/11/2011
MN877031/EEV-6/ZAF/EV130525 EP01992/1992
KF483800/EEV-4/ZAF/E080010 05/2009
MN876981/EEV-4/ZAF/E110580/2011
MN877011/EEV-6/ZAF/E110316 1/2011
HQ630908/EEV-1/ZAF/Bryanston/1976
MN876891/EEV-3/ZAF/Gamil M9 71/1971
HQ630898/EEV-6/ZAF/Potchefstroom/1991
72
90
93
75
76
84
99
98
99
97
97
89
87
98
73
0.010

(e)

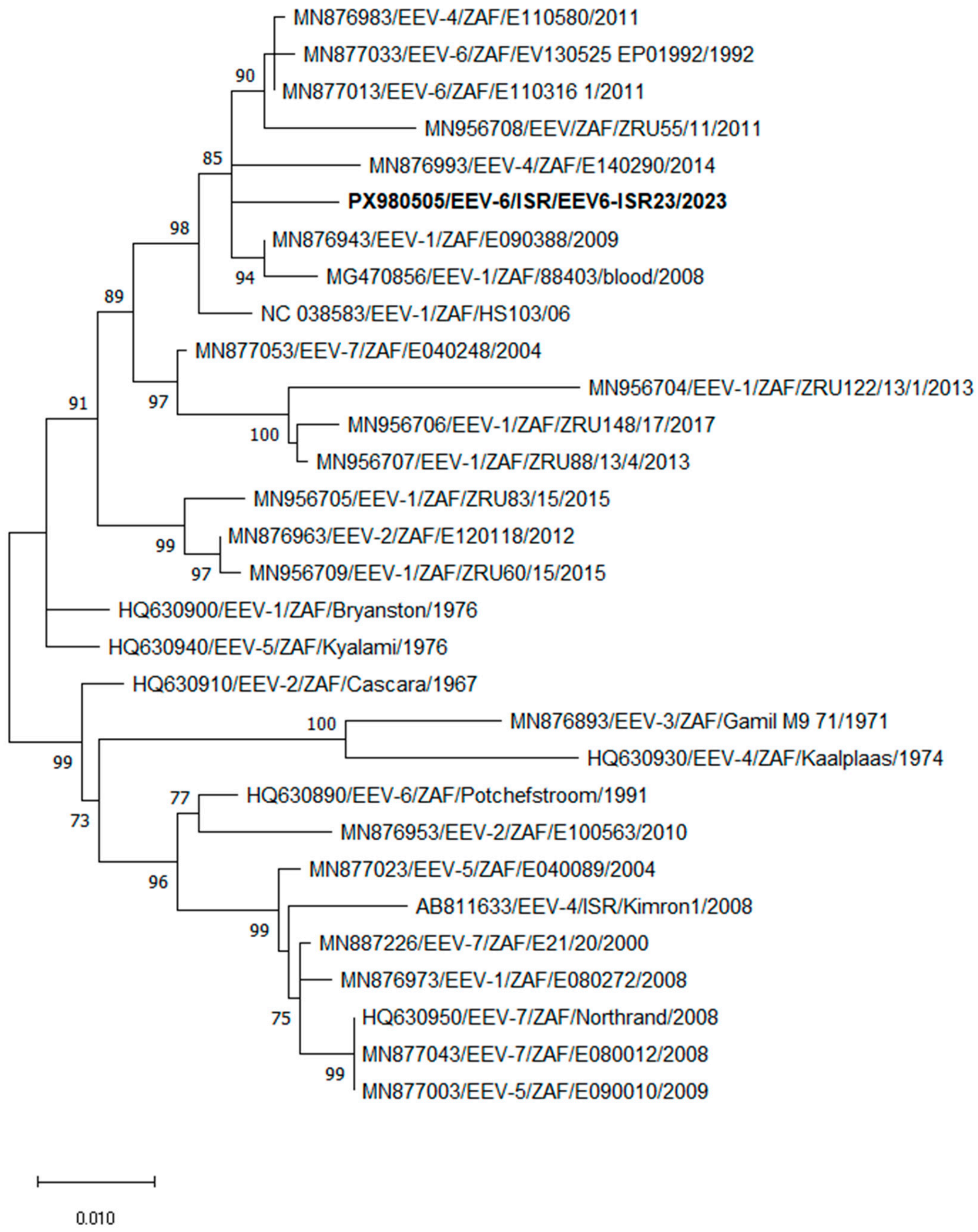

MN876983/EEV-4/ZAF/E110580/2011
MN877033/EEV-6/ZAF/EV130525 EP01992/1992
MN877013/EEV-6/ZAF/E110316 1/2011
MN956708/EEV/ZAF/ZRU55/11/2011
MN876993/EEV-4/ZAF/E140290/2014
PX980505/EEV-6/ISR/EEV6-ISR23/2023
MN876943/EEV-1/ZAF/E090388/2009
MG470856/EEV-1/ZAF/88403/blood/2008
NC 038583/EEV-1/ZAF/HS103/06
MN877053/EEV-7/ZAF/E040248/2004
MN956704/EEV-1/ZAF/ZRU122/13/1/2013
MN956706/EEV-1/ZAF/ZRU148/17/2017
MN956707/EEV-1/ZAF/ZRU88/13/4/2013
MN956705/EEV-1/ZAF/ZRU83/15/2015
MN876963/EEV-2/ZAF/E120118/2012
MN956709/EEV-1/ZAF/ZRU60/15/2015
HQ630900/EEV-1/ZAF/Bryanston/1976
HQ630940/EEV-5/ZAF/Kyalami/1976
HQ630910/EEV-2/ZAF/Cascara/1967
MN876893/EEV-3/ZAF/Gamil M9 71/1971
HQ630930/EEV-4/ZAF/Kaalplaas/1974
HQ630890/EEV-6/ZAF/Potchefstroom/1991
MN876953/EEV-2/ZAF/E100563/2010
MN877023/EEV-5/ZAF/E040089/2004
AB811633/EEV-4/ISR/Kimron1/2008
MN887226/EEV-7/ZAF/E21/20/2000
MN876973/EEV-1/ZAF/E080272/2008
HQ630950/EEV-7/ZAF/Northrand/2008
MN877043/EEV-7/ZAF/E080012/2008
MN877003/EEV-5/ZAF/E090010/2009
90
85
98
94
89
97
100
91
99
97
100
99
77
73
96
99
75
99
0.010

(f)

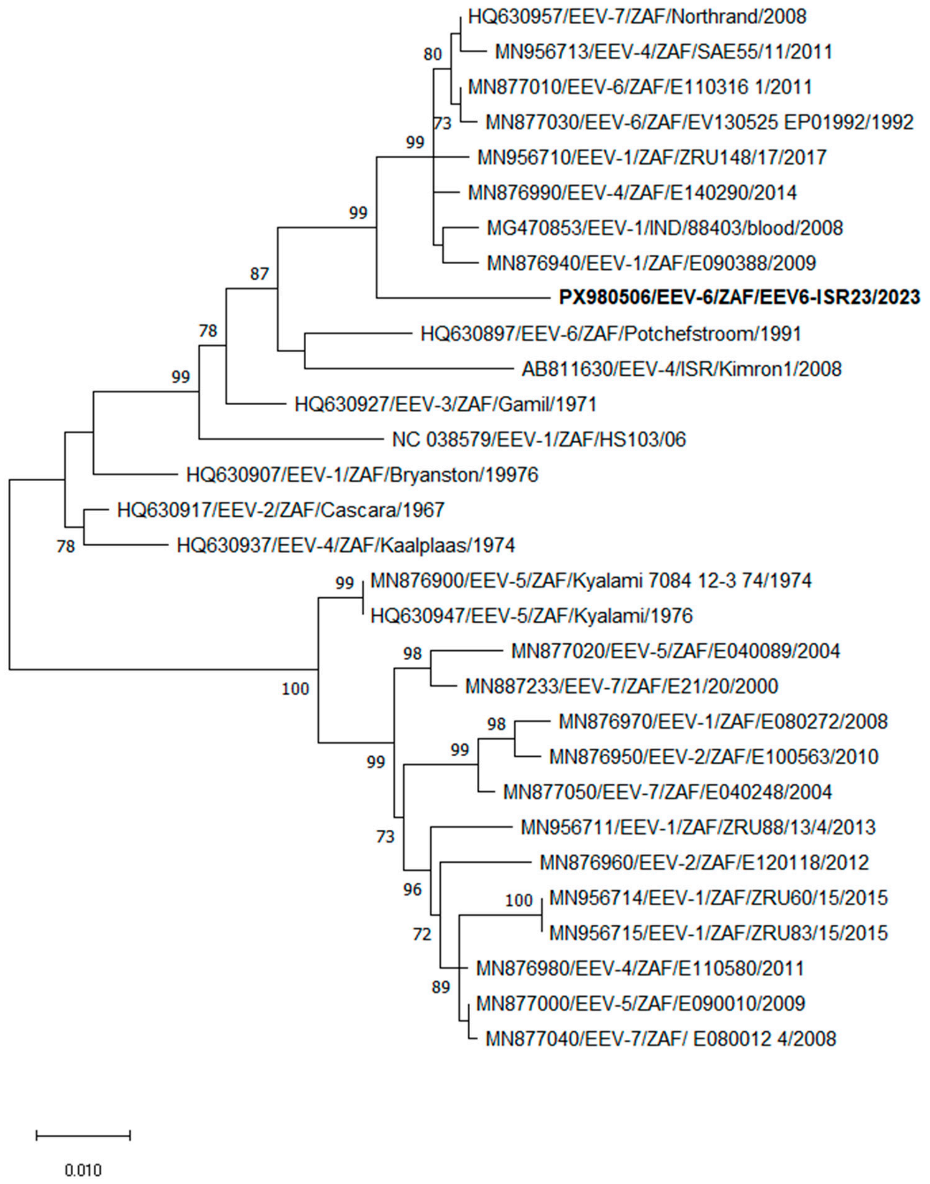
HQ630957/EEV-7/ZAF/Northrand/2008
MN956713/EEV-4/ZAF/SAE55/11/2011
MN877010/EEV-6/ZAF/E110316 1/2011
MN877030/EEV-6/ZAF/EV130525 EP01992/1992
MN956710/EEV-1/ZAF/ZRU148/17/2017
MN876990/EEV-4/ZAF/E140290/2014
MG470853/EEV-1/IND/88403/blood/2008
MN876940/EEV-1/ZAF/E090388/2009
PX980506/EEV-6/ZAF/EEV6-ISR23/2023
HQ630897/EEV-6/ZAF/Potchefstroom/1991
AB811630/EEV-4/ISR/Kimron1/2008
HQ630927/EEV-3/ZAF/Gamil/1971
NC 038579/EEV-1/ZAF/HS103/06
HQ630907/EEV-1/ZAF/Bryanston/19976
HQ630917/EEV-2/ZAF/Cascara/1967
HQ630937/EEV-4/ZAF/Kaalplaas/1974
MN876900/EEV-5/ZAF/Kyalami 7084 12-3 74/1974
HQ630947/EEV-5/ZAF/Kyalami/1976
MN877020/EEV-5/ZAF/E040089/2004
MN887233/EEV-7/ZAF/E21/20/2000
MN876970/EEV-1/ZAF/E080272/2008
MN876950/EEV-2/ZAF/E100563/2010
MN877050/EEV-7/ZAF/E040248/2004
MN956711/EEV-1/ZAF/ZRU88/13/4/2013
MN876960/EEV-2/ZAF/E120118/2012
MN956714/EEV-1/ZAF/ZRU60/15/2015
MN956715/EEV-1/ZAF/ZRU83/15/2015
MN876980/EEV-4/ZAF/E110580/2011
MN877000/EEV-5/ZAF/E090010/2009
MN877040/EEV-7/ZAF/ E080012 4/2008
80
73
99
99
87
78
99
78
99
100
98
98
99
99
73
96
72
100
89
0.010

(g)

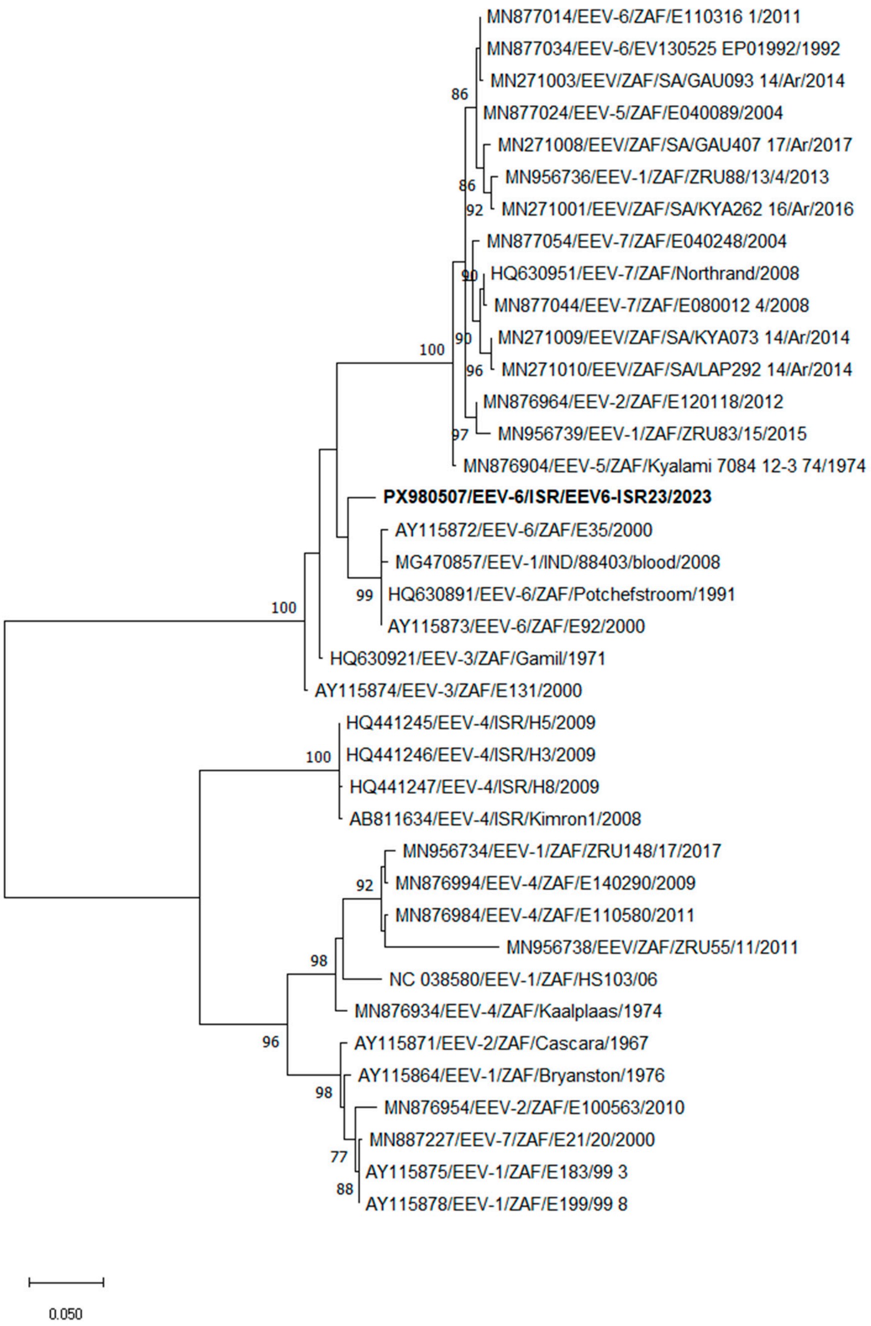

(h)

Figure S1. Phylogenetic trees of Israeli EEV-6 of internal genes and global strains. a) segment 1; b) segment 3; c) segment 4; d) segment 5; e) segment 7; f) segment 8; g) segment 9; h) segment 10. Israeli EEV-6 strain is shown in bold. The phylogeny was inferred using the Maximum Likelihood method and Tamura-Nei model method. The percentage of replicate trees in which the associated taxa clustered together in the bootstrap test (1000 replicates) are shown next to the branches. Only bootstrap values above 70% are displayed. Reference strains included all available strains from GenBank excluding duplicate sequences sharing nearly 100% nt identity, or sequences from the same outbreak sharing up to 100% of nt identity. Viruses were identified by accession number/serotype/location/isolate/year.
